# Linkage of community composition and function over short response time in anaerobic digestion systems with food fermentation wastewater

**DOI:** 10.1016/j.isci.2021.102958

**Published:** 2021-08-08

**Authors:** Weiwei Cai, Mingxing Zhao, Jianyao Kong, Silvio Riggio, Tim Finnigan, David Stuckey, Miao Guo

**Affiliations:** 1 School of Civil Engineering, Beijing Jiaotong University, Beijing 100044, China; 2 Department of Engineering, King's College London, London WC2R 2LS, UK; 3 Department of Civil and Environmental Engineering, Imperial College London, London SW7 2AZ, UK; 4 Department of Chemical Engineering, Imperial College London, London SW7 2AZ, UK; 5 School of Environment and Civil Engineering, Jiangnan University, Wuxi, Jiangsu Province, China; 6 Quorn Foods, Station Road, Stokesley, North Yorkshire TS9 7AB, UK

**Keywords:** Biological sciences, Biomass, Biotechnology, Chemical engineering, Microbiology

## Abstract

We investigated the short-term dynamics of microbial composition and function in bioreactors with inocula collected from full-scale and laboratory-based anaerobic digestion (AD) systems. The Bray-Curtis dissimilarity of both inocula was approximately 10% of the predicted Kyoto Encyclopedia of Genes and Genomes pathway and 40% of the taxonomic composition and yet resulted in a similar performance in methane production, implying that the variation of community composition may be decoupled from performance. However, the significant correlation of volatile fatty acids with taxonomic variation suggested that the pathways of AD could be different because of the varying genus. The predicted function of the significantly varying genus was mostly related to fermentation, which strengthened the conclusion that most microbial variation occurred within the fermentative species and led to alternative routes to result in similar methane production in methanogenic bioreactors. This finding sheds some light on the understanding of AD community regulation, which depends on the aims to recover intermediates or methane.

## Highlights

• The variation of the community was decoupled from AD performance
• The fermentative intermediates affect AD pathway rather than the AD performance
• The redundancy of fermentative species induces the independent community variation

## Graphical abstract

**Figure:**
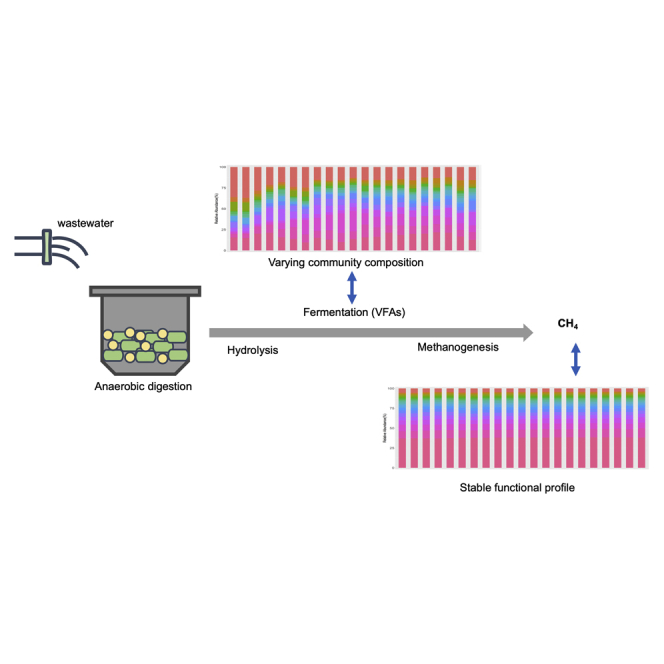

## Introduction

Microorganisms and their surrounding environments are the basis for a range of bioreactors, e.g. aerobic activated sludge, anaerobic digestion (AD), enabling the treatment of carbon or nutrient-enriched wastewaters; recently, increasing research attention has been paid to advancing the understanding of the functional role of microorganisms in bioreactors (Rittmann and McCarty, 2012). Culture-dependent characterization was commonly used to understand species functionality and how it influenced reactor performance (Roest, 2007; Vilela et al., 2020; Wagner et al., 1993). However, the core community of a bioreactor represents an artificial ecosystem consisting of multiple-syntrophic microbial communities, and it remains a significant challenge to understand the hidden mechanisms underlying the microbial ecology of bioreactors. Despite the ongoing controversy of the “1% culturability paradigm,” a majority of microorganisms in bioreactors, including specific functional species, may still be unculturable (Martiny, 2019, 2020; Steen et al., 2019). The rapid development of sequencing technology in the last 20 years has revealed an enormous microbial diversity; this has emerged as a culture-independent method to explore the ecological mechanisms underlying bioreactor microbial communities, advancing the understanding of hidden microbial ecological systems underpinning reactor performance (Rittmann et al., 2006).

The research questions about ecosystem function versus community composition in bioreactors opened up a Pandora's box: how do the microbial communities evolve, and how can the productivity and functional stability of a reactor be achieved or sustained (Fernández et al., 1999). Next-generation sequencing accelerated the discoveries in this area by generating new knowledge and understating at a molecular biology level. Advanced metagenomic sequencing (targeted 16s rRNA amplicons and/or shotgun sequencing) offers insights into taxonomic classification, microbiome composition, and powerful tools to monitor the AD process performances and inform operators how to optimize AD pathways and performance by regulating the community composition in bioreactors. This could lead to sophisticated bioaugmentation strategies and enhanced performance and stability. Despite the microbial ecology focus on coupling the community composition variance to their function in natural ecosystems (Waldrop et al., 2000; Waldrop and Firestone, 2006), a “decoupling” phenomenon existed. Notably, a long-term experimental study with amplicon and metagenomic sequencing showed that the microbial assembly relied on functional genes rather than species in accordance with the differences in Bray-Curtis distance (Burke et al., 2011). This perspective has been enhanced in further studies which considered the variation of community assemblages and associated functions (Louca et al, 2016, 2018). Generally, the community composition and functions are always interlinked because of the presence of functional species, which are often regarded as a performance index, or an indicator to predict the physiological dynamics of sludge, e.g. bulking and foaming (Wagner et al., 2002; Wagner and Loy, 2002; Yang et al., 2011). Several previous studies used the 16S rRNA or metagenomic sequencing technology to show the decoupling relationship between the community composition and the performance stability in artificial bioreactors in the presence of functional redundancy (Fernández et al., 1999; Fernandez-Gonzalez et al., 2016; Vanwonterghem et al., 2016; Wang et al., 2011; Wittebolle et al., 2008). Recent studies showed that the functional redundancy of Fe(II) metabolism impacted the functional stability under a wide range of pH and Fe(II) concentrations (Ayala-Muñoz et al., n.d.). Although functional redundancy was considered as the driver for this decoupling, recent research has provided a different perspective showing that strong links exist between community composition and function, which disagrees with the redundancy widely observed in marine environments (Galand et al., 2018). Interestingly, strong correlations between community composition and function have been also demonstrated in previous research where the microbial community was applied as a training database to predict bioreactor performance (Günther et al., 2012; Lesnik and Liu, 2017). Overall, the relationship between community composition and function in bioreactors remains as a research frontier worthy of more in-depth exploration. Notably, limited studies have been published in this field investigating AD with industrial wastewater, with a notable gap on food-fermentation wastewater.

In this study, experiments were performed in continuous stirred-tank reactors (CSTRs) to investigate changing and community composition during reactor start-up with a carbon-rich wastewater generated from the fermentation industry. Quorn Foods was selected to represent the advanced fermentation technology, and wastewater was collected from a mycoprotein production process at Quorn which are currently aerobically treated on-site. We have investigated different inocula originating from a full-scale reactor (inocula-F) and a laboratory-based system (inocula-L). The former was obtained from a centralized full-scale AD plant codigesting wastewater and organic solid waste, while the latter was obtained from a laboratory-scale anaerobic membrane bioreactor described in a previous study (Tao et al., 2020). We selected the classical CSTR as the AD reactor in this study; a preadaptation was used to acclimate the inocula to adapt to the unique food-fermentation industrial wastewater from a Quorn mycoprotein production process. The same environmental stressor (ecological factor) was applied on different inocula that represent the distinct microbial sources; the similar performance observed from a CSTR offers evidence to elucidate that the community variation could be decoupled from the reactor performance over a short-term response period. Thus, the parameters for all CSTR were the same, which were also the key to compare the reactor performance with different inocula.

Specifically, the two different inocula were preacclimated in batch reactors for 42 days (d) followed by a start-up of CSTRs inoculated with these acclimated sludges. All samples were collected daily during the preacclimation experiment and the start-up period of the CSTRs (23 d). The water samples were characterized by analytical methods to determine chemical oxygen demand (COD), volatile fatty acids (VFAs), total suspended solids (TSS), and volatile suspended solids (VSS). Biosamples were prepared for amplicon sequencing analysis following the DNA extraction and sequencing protocol detailed in the STAR Methods section. Overall, this study tested the hypothesis that the functional stability of AD over a short-term response period in anaerobic bioreactors fed with food-fermentation industry wastewater could be decoupled from the community composition variance because of the presence of functional redundancy, while the community composition variance may have an impact on the intermediate's generation. Two aspects enabled us to investigate the relationship between community variation and reactor performance, i.e. different inocula and the variation of specific community in a short term in anaerobic bioreactors. The former reflected the variance in microbial sources and initial composition, whereas the latter focused on the stability of reactor performance over a short-term community variation.

## Results and discussion

### Performance of reactors: preacclimation

The initial inocula collected from the wastewater treatment systems (inocula-F and inocula-L) were introduced into Automated Methane Potential Test System (AMPTS) batch reactors to characterize the initial activity of these inocula under preacclimation experiments. As shown in Figure S1 and Table S1, similar daily methane production trends were found across samples with varying hydraulic retention times (HRTs). The methane production of each cycle duration (4 d for inocula-F and 6 d for inocula-L) was 241.35 ± 15.23 NmL CH_4_ for inocula-L and 217.74 ± 40.48 NmL CH_4_ for inocula-F, indicating significantly higher performance of inocula-L than that of inocula-F (t test, p < 0.05). The inocula-F was originally collected from a wastewater treatment plant with inert and nonbiodegradable organics, which may reduce the activity of microorganisms per mass of culture. Considering the removal of COD each day, the average methane yield of inocula-L was 7.55 ± 2.05 NmL CH_4_/g COD_[removed]_, which was lower than the inocula-F average (9.41 ± 2.24 NmL CH_4_/g COD), but these yields were not statistically different (p > 0.05).

### Performance of reactors: start-up and operation

**Figure 1:**
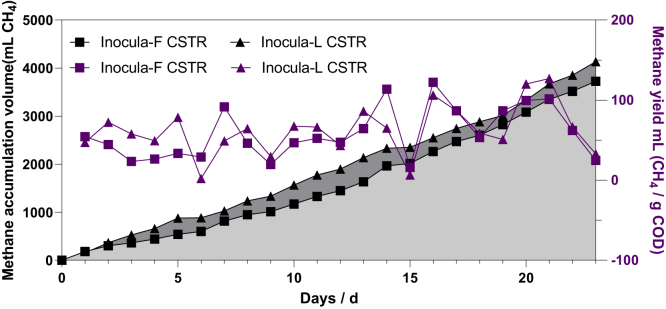
Operational performance of inocula-F and inocula-L CSTR; the rectangle represents the results of inocula-F, the triangle represents the results of inocula-L, the purple color represents the methane yield, and the black color represents the methane accumulation volume

The preacclimated sludges were inoculated into the CSTRs to obtain stable methane production. As shown in Figure 1, the average methane production was 161.96 ± 76.11 NmL CH_4_/d and 179.71 ± 80.04 NmL CH_4_/d for inocula-F and inocula-L, respectively (p > 0.05). The COD removal efficiency was in the range of 30%–50% over the 23 days of operation, averaging 37.64 ± 7.01% for inocula-F, which was close to the performance of inocula-L (36.42 ± 5.85% COD removal, p > 0.05). As a similar amount of organics had been removed during methane generation, there was no significant difference in the observed methane yield (p > 0.05), and the CSTR inoculated with preacclimated inocula-F produced 58.51 ± 31.87 NmL CH_4_/g of COD daily, whereas inocula-L sludge generated 62.33 ± 30.75 NmL CH_4_/g COD. Although the original seed sludge was different in the two CSTRs, there was no statistical difference in their performance in terms of methane production and COD removal as the different inocula had evolved and improved over fed batch experiments, especially inocula-F.

A further specific methanogenic activity (SMA) test confirmed the similar capacities of inocula-F and inocula-L sludge. As shown in Figure S2, the cultured inocula-F sludge showed a similar performance (0.30 ± 0.08 g CH_4_/g VSS/d) to inocula-L (0.28 ± 0.11 g CH_4_/g VSS/d, p > 0.05).

**Figure 2:**
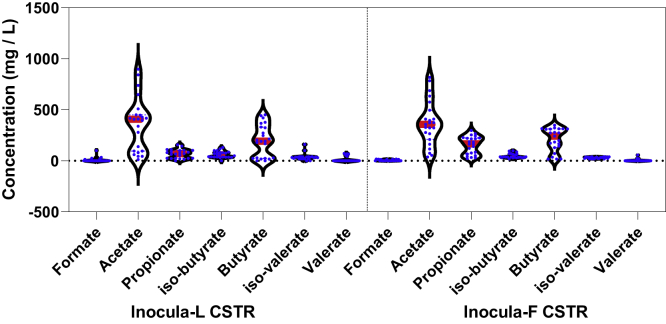
Violin plot of VFA concentrations in inocula-F and inocula-L CSTR; the stars represent significance (p < 0.05), the red line is the mean value of each fatty acid

A pH increase was observed in both CSTR reactors over time – from 7.5 to 7.88 for inocula-F and from 7.5 to 7.73 for inocula-L (Figure S3), although the decrease of pH in the first days could be caused by the accumulation of VFAs (Figure S4). The fluctuation in pH was significantly different (p < 0.05), where inocula-F and inocula-L averaged 7.66 ± 0.25 and 7.56 ± 0.19, respectively. The variation in VFAs exhibited visible differences in Figure 2. The propionate concentration in inocula-L was significantly higher than that of inocula-F (p < 0.05); in contrast, significantly lower iso-butyrate concentrations were found in inocula-L CSTR than in inocula-F (p < 0.05). As shown in Figure S4, the change of VFAs in inocula-F and inocula-L CSTR was clearly different. In AD, VFA production from complex organics would eventually be converted into acetate and hydrogen which are the main substrates for methanogenesis, although the dynamics of VFA production are influenced by thermodynamic limitations caused by high-hydrogen partial pressure; therefore, the conversion pathways of VFAs could be diverse (Łukajtis et al., 2018). Despite the similarity of performance in methane production, the variation in VFAs suggested that the fermentative pathway of the microbial community could be different for inocula-F and inocula-L CSTRs.

**Figure 3:**
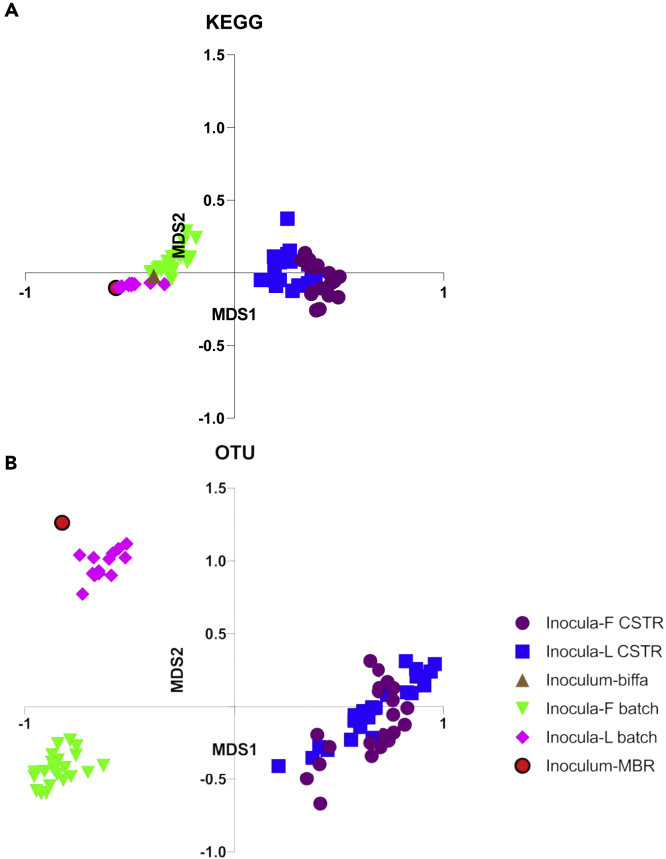
A clustering results of all samples MDS plots of KEGG (A) and OTU (B) based on Bray-Curtis distance.

**Figure 4:**
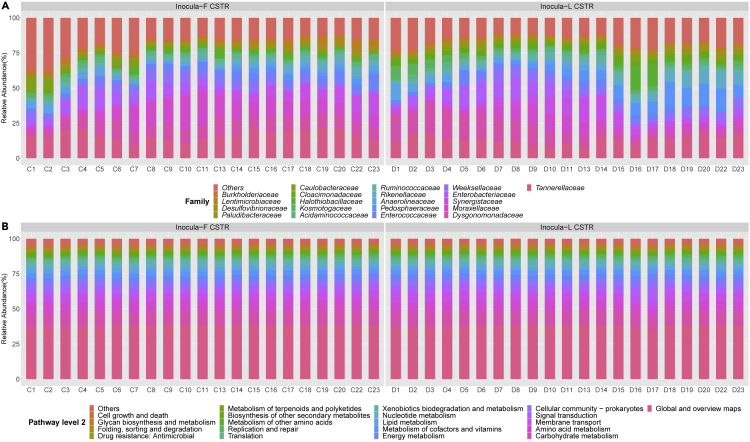
Microbial taxonomy for CSTR stage Variation of microbial composition at genus level (A) and KEGG pathway 2 level (B).

**Figure 5:**
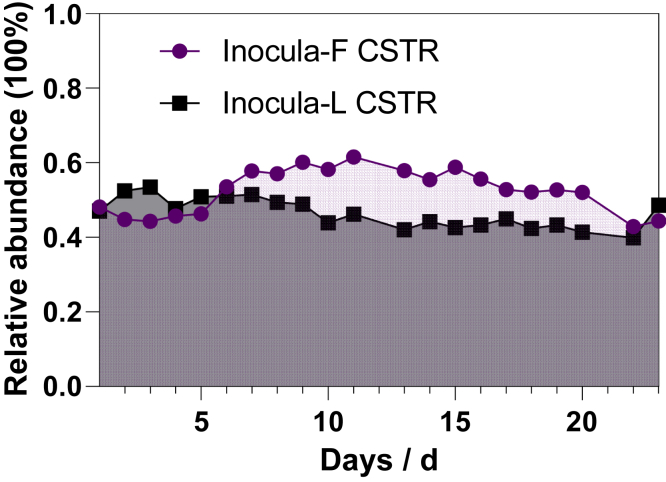
The variation of relative abundance of fermentative microorganisms over the operational period of the CSTR

### Variation of microbial composition and function profile

The microbial taxonomy initially present in inocula-F and inocula-L sludge differed; *Mesotoga* accounts for nearly 80.70% and 31.20% in inocula-L and inocula-F sludge (Figure S5), respectively, resulting in a clear difference of diversity (Shannon index, 1.40 vs 3.34, shown in Figure S6). The subsequent preacclimation eliminated the dominant advantage of *Mesotoga*, which declined to 53.80% ± 17.60% and 17.30 ± 6.60% at the end of the batch cycle. As shown in Figure S6, the Shannon index increased in the batch mode of both inoculated reactors, considering the microbial evolution, while the phylogenetic diversity (PD) was steady over the batch operational period (Figure S7). The PD of inocula-F and inocula-L was 9.84 ± 0.23 and 8.55 ± 0.44, respectively (p < 0.05). The PD value of inocula-F was still higher than that of inocula-L, which accorded with the alpha diversity index. There is a slight change of PD value in both inocula over the adaptation period, implying the change of microbial community was insignificant.

In the continuous mode (shown in Figure S8), the phylogenetic diversity decreased over time in both the CSTRs inoculated with full-scale and laboratory sludge. The microbial community of inocula-F evolved in the CSTR with a significantly higher PD compared to inocula-L (p < 0.05). The PD value has been previously shown to be relevant to the stability of reactors, as the diversity provided a broader metabolic potential to adapt to environmental shocks in wastewater treatment bioreactors (Yang et al., 2011; Zhang et al., 2019). From the viewpoint of microbial ecology, the species as phylogenetic relatives could compete for resources with a similar metabolism, and a lower PD value indicates that most species evolved from the same ancestor with a similar metabolism; however, in contrast, a higher PD may reflect complex metabolic patterns (Cordero et al., 2012; David et al., 2014). The preacclimation enabled the cultures to maintain the PD values which would be beneficial for a community adapting to the environment. However, the washout and enrichment in the CSTRs indicated that a specifically functional unit would be enhanced with decreasing PD, which commonly occurred in our previous studies (Cai et al., 2016, Cai et al., 2019, Cai et al., 2020).

A variation in community composition was revealed by the change in PD; a clustering result is depicted in Figure 3A where the nonmetric multidimensional scaling (NMDS) based on the Bray-Curtis distance demonstrated that the inocula-F batch was far away from the cluster of inocula-L batch, and the difference of operational taxonomic units (OTU) composition contributes to this distance. The corresponding Bray-Curtis dissimilarity between inocula-F and inocula-L batches was 75.96% ± 4.72%. The scatters representing inocula-F CSTR and inocula-L CSTR were clustered together; if considering time variation, the position of the time-dependent plots distributed from left to right on the main axis and from bottom to top in the second axis. The averaged OTU-based dissimilarity between the inocula-F and inocula-L CSTRs was 41.60% ± 5.40%, which was nearly half that in the batch mode. In addition, the Kyoto Encyclopedia of Genes and Genomes (KEGG) pathways (level 3) predicted by Tax4fun2 exhibited different clustering results of NMDS and Bray-Curtis dissimilarity in contrast to OTU-based analyses; as shown in Figure 3, inocula-F and inocula-L batches were clustered together with lower dissimilarity (18.39 ± 4.57%). However, an average dissimilarity of 14.23 ± 3.69% was found throughout the operation of the CSTRs.

The difference in distance between the OTU and KEGG matrices suggests that the function dynamic was different from OTU variation, i.e., a stable function might be obtained with varying composition; this research finding is in accordance with a previous study by Burke et al. Considering the time effect, as shown in Figure S9, there is no observed trend in CSTR dissimilarity over time, but the stationary check augmented Dicky-Fuller test (ADF) verified that the change in OTU-based and KEGG-based Bray-Curtis dissimilarity is nonstationary, i.e., time relevant (both p > 0.05). The positive coefficient of linear regression indicated an invisible increased trend of dissimilarity for OTU-based and KEGG-based dissimilarity (0.0027 for OTU and 0.0003 for KEGG). As shown in Figure 4, the variation of genus was clear, whereas the fluctuation in the KEGG pathway level 2 was invisible. These results were in accordance with the Bray-Curtis dissimilarity as the function profile was stable over the operational period.

### Environmentally driven changes in community assembly

The contribution of environmental factors to community assembly was summarized as a deterministic theory, which is in contrast to stochastic or neutral processes (Zhou et al., 2013). The null model can quantify the dominance of each process by adopting the index of normalized stochasticity ratio (NST). Clearly, the community assembly was more stochastic for inocula-F (72.17%) than the stochastic assembly of inocula-L CSTR (87.67%), indicating the shift in community could be mostly explained by stochastic processes rather than environmental determinism; in addition, a higher proportion of neutral processes was commonly found in previous studies (Zhou et al., 2013). The redundancy of the microbial community was determined by redundancy analysis (RDA). The environmental variables explained 57.40% and 52.90% for inocula-F and inocula-L group, respectively. As mentioned earlier, functional redundancy could exist in a phylogenetically diverse community, while stochasticity could be dominant in modulating the redundancy section of the community without negative effects on performance.

The partial Mantel test based on Bray-Curtis dissimilarity was used to detect the correlation between environmental variables and the microbial community. As shown in Table 1, the pH significantly affected the variations in the inocula-F community at the OTU level (r = 0.72, p < 0.05), as well as the inocula-L CSTR system (r = 0.46, p < 0.05), while the iso-butyrate and iso-valerate were significantly correlated with community variation in inocula-F CSTR (r = 0.24, p < 0.05 and r = 0.40, p < 0.05). The iso-butyrate was also significantly correlated with community fluctuation (r = 0.28, p < 0.05) in the inocula-L CSTR. In addition, the acetate and propionate only demonstrated significant association (r = 0.19, p < 0.05 for acetate and r = 0.15, p < 0.05 for propionate) in inocula-L CSTR. Considering the variations in the KEGG pathway (level 3), pH was still significantly correlated with the inocula-F community in the CSTR, as the partial Mantel is 0.64 (p < 0.05). However, inocula-L CSTR showed an insignificant correlation with pH (p > 0.05), while the significant factors that affected the change of the KEGG pathway were propionate (r = 0.31, p < 0.05) and COD (r = 0.44, p < 0.05) in the inocula-L CSTR. The results of iso-butyrate and iso-valerate from the inocula-F CSTR were still significant, r = 0.31 (p = 0.003) and r = 0.31 (p < 0.05), respectively. Overall, performance was similar between the inocula-F and inocula-L CSTRs, which could be ascribed to their stable functional profiles even under the presence of taxonomic variation. Although the correlation pattern of VFAs with taxonomic variation were different between inocula-F and inocula-L CSTR, the community variation showed a strong correlation with metabolic products in both. The metabolic products could be affected by the microbial taxonomy, leading to a significant difference in VFA concentrations.

**Table 1:** Partial Mantel test results of inocula-F and inocula-L in the CSTRs

Environmental factors	Inocula-F				Inocula-L			
	OTU level		KEGG level		OTU level		KEGG level	
	r	p	r	p	r	p	r	p
pH	0.7172∗	0.001	0.641∗	0.001	0.4637∗	0.002	0.0824	0.139
Formic	0.1232	0.137	0.0225	0.372	0.091	0.124	−0.0828	0.789
Acetic	−0.2862	1	−0.2166	0.986	0.1933	0.022	0.0015	0.447
Propionic	−0.2292	0.993	−0.1542	0.958	0.1472∗	0.034	0.3077∗	0.001
Isobutyric	0.2357∗	0.02	0.3063∗	0.003	0.2757∗	0.005	0.0428	0.269
Butyric	−0.2424	0.999	−0.2325	1	−0.1597	1	−0.0485	0.701
Isovaleric	0.4048∗	0.004	0.3069∗	0.006	0.1109	0.071	0.0162	0.455
Valeric	−0.2462	0.999	−0.1665	0.973	−0.0852	0.837	−0.0985	0.803
COD	0.0515	0.304	0.0413	0.311	0.1271	0.1	0.4429∗	0.004
Methane	−0.0278	0.547	−0.0185	0.529	−0.0748	0.816	−0.1535	0.942

∗represent the significant result.

The functional profile of the microbial communities was constructed by referring to Midas, as well as to FAPROTAX and previous literature, and the results can be found in Table S2 and Figure 5. A significantly varying genus was defined as those which showed significant variance using the one sample t test, and the sum of relative abundance of varying species reached more than 80% in inocula-F or inocula-L group.

A shown in Figure S9, the most significantly varying genus had a metabolism which underpinned the fermentation process, accounting for 52.48 ± 5.91% for inocula-F and 46.42 ± 4.05% for inocula-L. In AD, the initial compounds are catabolized through a range of biochemical pathways including hydrolysis, fermentation, acetogenesis, and methanogenesis to achieve energy recovery and organics removal. The species-related VFAs always show high diversity in the phyla of *Bacteriodates*, *Chloroflexi*, *Firmicute*, etc., as their functional redundancy provided alternative routes for VFA production (De Vrieze et al., 2015; Zamanzadeh et al., 2016). Thermodynamic feasibility favors diversity as the free energy associated with the endpoint of linear catabolic reactions was nonsensitive to taxonomic variation with the same electron donor and acceptor (Rittmann and McCarty, 2012). Detailed modeling of a methanogenic bioreactor reported in previous research confirmed that the functional redundancy is related to taxonomic variation and increases the functional stability in response to phage invasion (Louca and Doebeli, 2017).

In addition to fermenters, the relative abundance of methanogens was relatively stable without significant variation except *Methanobacterium* in inocula-L CSTR. The stable methanogenic population could be due to the fact that methanogens only utilize a few substrates (hydrogen, formate, acetate, or methyl) to generate methane and are categorized into three groups of hydrogenotrophs, acetotrophs, and methylotrophs, which are comprised of several classes (*Methanobacteria*, *Methanococci*, *Methanomicrobia*, *Methanonatronarchaeia,* and *Methanopyri*) (Baker et al., 2020). The functional redundancy was rarely observable by a significant variation in the methanogenic community in the anaerobic digester, i.e., the methanogens detected were stable as a few genera of methanogens over time in the methanogenic bioreactors (Tao et al., 2020). The generally dominant methanogens were *Methanosarcina*, *Methanosaeta*, and *Methanobacterium* (Demirel and Scherer, 2008). On the other hand, we believe that the ecological niches of methanogens may be phylogenetically diverse, as species from the order Methanomicrobiales fall in the pH range of 5.1–7.4 and generation time from 10 hrs to 144 hrs (Browne et al., 2017); hence, the unique AD environment seems to lead to a specific methanogenic community. The correlation between VFAs and community variation also implies that the fermentation stage has broadened functions associated with a taxonomic shift. Iso-butyrate was found to be the driving factor which impacts the digestion process of inocula-F wastewater. Overall, an outline of the relationship between performance and composition variation gradually emerged from our work as the taxonomic fluctuation of fermenters could be decoupled from the variation in performance, which relied on the presence of redundancy, while coupled with the variation of metabolic products during the fermentation.

In summary, the decoupling of taxonomic variation and function in methane production was observed, concluding that taxonomic variation may not be sufficient for predicting the function of a specific bioreactors. Further analysis indicated that the redundancy in the fermenters is likely to be the key to achieve such decoupling. Importantly, the VFAs varied substantially over the period of operation (time), which was coupled to a high proportion of fermentative species varying significantly, implying that taxonomic variation changed the pathway of fermentation while resulting in similar functional profiles. Overall, our research contributed insights into microbial ecology in AD, for both research communities and industry practitioners. In particular, our study highlighted research directions needed on functional variation rather than taxonomic changes in microbial communities when analyzing AD reactors. Specifically, our research suggested that the redundancy of fermenters diversified the metabolic pathway to a similar methane production in anaerobic bioreactors; our observation implied that a high-throughout functional genes variation could be a more insightful indicator to underpin the reactor performance, in comparison with 16S rRNA gene sequencing with highly diverse taxonomy.

### Limitations of the study

Although the experimental analysis showed the function variation could be decoupled from microbial community changing, a long-term monitor of functional genes will provide solid proof on the stable function in specific metabolism flow. A further study focused on the specific metabolism variation in AD will provide advanced understanding in the coupling of function and microbial community.

## STAR★Methods

### Key resources table

**Table:** 

REAGENT or RESOURCE	SOURCE	IDENTIFIER
Software and Algorithms		

R software	https://www.r-project.org/	Version 3.6
R studio (Open Source License)	https://www.rstudio.com/	Version 1.4.1717

### Resource availability

#### Lead contact

Further information and requests for resources and reagents should be directed to and will be fulfilled by the Lead Contact, M.G (miao.guo@kcl.ac.uk).

#### Materials availability

This study did not generate new unique reagents.

#### Data and code availability

• All data sets are available in supplemental information. All raw sequencing data have been deposited in the National Center for Biotechnology Information (NCBI) as the reference number of PRJNA678675, and it can be accessed now at NCBI.
• This study did not generate original code.
• Any additional information required to reanalyze the data reported in this work is available from the Lead Contact upon request.

### Method details

#### Experimental setup

The inocula collected from the different AD systems were preacclimated prior to inoculating the CSTRs to treat fermentation wastewater from the food industry. To enable potentially different microbial ecologies to be studied, different inocula were collected from a bench-scale laboratory anaerobic membrane bioreactor (inocula-L) and a UK commercial AD plant codigesting the biodegradable fraction of municipal solid waste and municipal wastewater (inocula-F), which operated mesophilic (30-40°C), wet (< 15% dry solid), continuous-feeding, and multiple stage (hydrolysis step and methanogenesis stage) digestion systems. In this research, Quorn Foods was selected to represent the advanced fermentation technology which generates carbon-rich wastewater. Wastewater samples were collected from the mycoprotein production process at Quorn which are currently treated on-site to meet environmental regulations. However, the fermentation wastewater represents carbon- and nutrient-rich resources which can be potentially recovered as value-added products. The preacclimation of these two inocula was operated for 42 d followed by a start-up of CSTRs inoculated with these acclimated sludges. All samples were collected daily during the preacclimation experiment and the start-up period of the CSTRs (23 d). The water samples were characterized by conventional analytical methods, while the biosamples were prepared for amplicon sequencing analysis.

#### Preacclimation experiment

Inocula-F and inocula-L were preacclimated to the fermentation wastewater using an AMPTS (Bioprocess Control Sweden AB, Sweden), where the inocula collected from both reactor systems were compared. The preacclimation assay was conducted in reaction bottles with a total liquid volume of 400 ml and a 100-ml headspace. Anaerobic conditions were maintained during inoculum and substrate transfers by flushing the reaction bottles with N_2_ at a flow rate of approximately 0.5 L/min. All reaction bottles were incubated at 35°C at an initial pH of 7.5, stirring at 200 rpm. The initial organic loading rate was 2 kg COD/m^3^ per day.

The AMPTS was operated in batch mode, and inocula preacclimation was achieved after 7 adaptation cycles (fed batch). The adaptation cycle duration for the two inocula followed before experiments where the cumulative biogas production reached a plateau, and these cycles were determined to be 4 and 6 days, for inocula-F and inocula-L, respectively. At the end of each cycle, supernatant from each reaction bottle was removed and replaced by new fermentation wastewater feed. For each adaptation cycle, the liquid samples were collected to determine the concentration of VFAs and COD, while 2 ml of biomass samples was collected for DNA extraction and 16s rRNA gene sequencing.

#### Continuous stirred-tank reactors

Two CSTRs with an effective reaction volume of 1.7 L (2 L of total volume) were inoculated with the two preacclimated sludge samples. The reactors were glass cylinders as shown in Figure S11. Fermentation wastewater was continuously fed into the CSTRs at an organic loading rate of 2 kg COD/m^3^/day, and a ratio between VSS and soluble chemical oxygen demand (SCOD) of 1:1. The CSTRs were run at room temperature and HRT of 2 days, stirring at 100 rpm by an orbital mixer, while the pH of the influent wastewater was maintained at 7.5. The CSTRs were connected to the AMPTS system to monitor the biogas yields. Samples were collected daily from the CSTR effluent for VFA and COD analyses, while biomass samples were collected from the CSTRs for DNA extraction and 16s rRNA gene sequencing, as in the preacclimation experiment.

#### Analytical methods

SMA was used to estimate the activity of the methanogenic microorganisms to convert acetate into biomethane. The SMA of the two inocula was determined using an AMPTS with a VSS concentration of 2 g/L. To ensure that we reached a saturation level of substrate (>2K_s_), acetic acid at two concentrations of 1.2 g/L and 1.6 g/L was used for the SMA assays. The SMA was expressed as kg CH_4_/COD/kg VSS/day (Tao et al., 2020).

COD was measured using a COD kit (Lovibond). Vials with 0-1,500 mg/L and 0-15,000 mg/L were used depending on the sample concentrations; a sample volume of 2 mL was added to the vial at low range measurements, and 0.2 mL at high range measurements. After the tubes were sealed and inverted three times (to mix properly), they were placed in a COD thermoreactor (Lovibond RD125) at 150°C for 2 hours. The samples were analyzed on a UV/VIS scanning spectrophotometer (Lovibond MD 200) at 600 nm wavelength.

VFAs and the suspension samples collected from the AMPTS and CSTRs were filtered through a 0.22-μm filter (VWR Labshop). Chromatography analysis of the supernatant was carried out using a Shimadzu HPLC system with UV/Vis detectors (210/254 nm) and calibrated to detect formate, acetate, propionate, iso-butyrate, butyrate, iso-valerate, and valerate. The HPLC was equipped with a Bio Rad Aminex HPX-87H column measuring 300 x 7.8 mm and set at 55°C. The mobile phase used was a 0.005 M H_2_SO_4_ solution, and its flow was set at 0.6 ml/min. The injected volume was 10 uL, and the analysis time was 35 min.

TSS and VSS were assayed according to standard methods (APHA, 1998). A small sample (0.1-0.25 mL) was transferred to a filter and rinsed with deionized water three times. The filter with sample was transferred to an aluminum dish and oven-dried at 103-105°C for 24 hours to determine TSS. The dish and filter were then placed in the muffle furnace at 575 ^º^C overnight and weighted to determine VSS.

#### DNA extraction and sequencing procedure and analysis

DNA in the biomass samples collected from the CSTRs and AMPTS were extracted using the DNeasy PowerSoil microbial DNA isolation kit (QIAGEN, Germany). DNA purity was verified by Nucleic Acid 260/280 Ratios using a Nanodrop Spectrophotometer 2000 (Thermo Scientific, USA). The amplification, library construction, and sequencing of the 16S rRNA gene were performed by The Earlham Institute using the following protocol. The generic primer (f: V4: GTGCCAGCMGCCGCGGTAA, r: V5: CCCGTCAATTCMTTTRAGT) was used to amplify the specific region V4 of the 16S rRNA gene to prepare for further sequencing. The subsequent sequencing was completed with a MiSeq Illumina Sequencing Platform following the method detailed in the study by Kozich et al., 2013. All raw sequencing data were processed online to filter and remove N bases, barcode, and primer (Feng et al., 2017). The filtered data were clustered by Unoise to generate OTUs, and the OTU table was classified by RDP classifier using the method of Silva (Quast et al., 2012). The OTU table was then normalized by 16S copy number according to the *rrn*DB (ribosomal RNA operon copy number database) (Stoddard et al., 2015). The adjusted OTU table was resampled to ensure equal total reads of each sample. Tax4fun2 prediction based on 16S sequencing data was completed with the R package (Aβhauer et al., 2015; Ihaka and Gentleman, 1996). All diversity index and PD values were calculated with vegan and picante R packages (Faith, 1992). The Bray-Curtis dissimilarity, Mantel test, and PD were completed online (http://mem.rcees.ac.cn:8080/root) (Feng et al., 2017), and other statistical analyses were performed using the R package (Ihaka and Gentleman, 1996). The Microbial Database for Activated Sludge (MiDAS) was used to predict the potential metabolic function of genus (McIlroy et al., 2017). The phylogenetic tree was visualized using the iTOL online tool (https://itol.embl.de/) (Letunic and Bork, 2016). All significant statistics were evaluated using a two-tailed t- test with paired samples. All average values were presented with a standard deviation (±SD). The NST was calculated using the R package NST (Ning et al., 2019). Database: PRJNA678675. All raw sequencing data have been deposited in the National Center for Biotechnology Information (NCBI) as the reference number of PRJNA678675, and it can be accessible now at NCBI (Leinonen et al., 2011).

## Supporting citations

The following references appear in the Supplemental information(Gautam et al., 2009; Jabari et al., 2012; Lawson et al., 2010; Nesbø et al., 2012; Schreiber et al., 2006; Ueki et al., 2006; Yabuuchi et al., 1983).

## Footnotes

Supplemental information can be found online at https://doi.org/10.1016/j.isci.2021.102958.
