# Supplementary material for: Linkage of community composition and function over short response time in anaerobic digestion systems with food fermentation wastewater
Source: iScience. 2021 Aug 8;24(9):102958. doi: 10.1016/j.isci.2021.102958 (PMC8384924; doi:10.1016/j.isci.2021.102958)
Supplement: Document S1. Figures S1–S11 and Tables S1 and S2 [file mmc1.pdf]

iScience, Volume *24*

Supplemental information

Linkage of community composition and function over short response time in anaerobic digestion systems with food fermentation wastewater

Weiwei Cai, Mingxing Zhao, Jianyao Kong, Silvio Riggio, Tim Finnigan, David Stuckey, and Miao Guo

Supplementary Information

The linkage between community composition and function over the short-term response period in anaerobic digestion systems with food-fermentation industrial wastewater

Weiwei Cai[1,2*7], Mingxing Zhao[3,4,5,7], Jianyao Kong [4], Silvio Riggio[4], Tim Finnigan[6], David Stuckey[4], Miao Guo[2,4,8*]

[1] School of Civil Engineering, Beijing Jiaotong University, Beijing, 100044, China.

[2] Department of Engineering, King's College London, WC2R 2LS, UK

[3] Department of Civil and Environmental Engineering, Imperial College London, SW7 2AZ, UK.

[4] Department of Chemical Engineering, Imperial College London, SW7 2AZ, UK

[5] School of Environment and Civil Engineering, Jiangnan University, China.

[6] Quorn Foods, Station Road, Stokesley, North Yorkshire TS9 7AB, UK

[7] These authors contributed equally

[8] Lead contact

[*] Corresponding author:

miao.guo@kcl.ac.uk (MG)

wwcai@bjtu.edu.cn (WWC)

Table S1 Bioreactor performance (Related to Figure 1)

Operational performance of inocula-F and inocula-L at the stage of adaptation and CSTR

	Adaptation				CSTR				
	HRT / d	VSS_{in}	COD Removal efficiency (%)	Methane production (mL / cycle)	HRT / d	VSS_{in}	pH	COD Removal efficiency (%)	Methane production (mL / d)
Inocula -F	4	2	93.94 ± 11.37	217.74 ± 40.48	2	2	7.66 ± 0.25	37.64 ± 7.01	173.82 ± 81.69
Inocula -L	6	2	99.12 ± 2.24	259.04±16.35	2	2	7.56 ± 0.19	36.42 ± 5.85%	192.88 ± 85.90

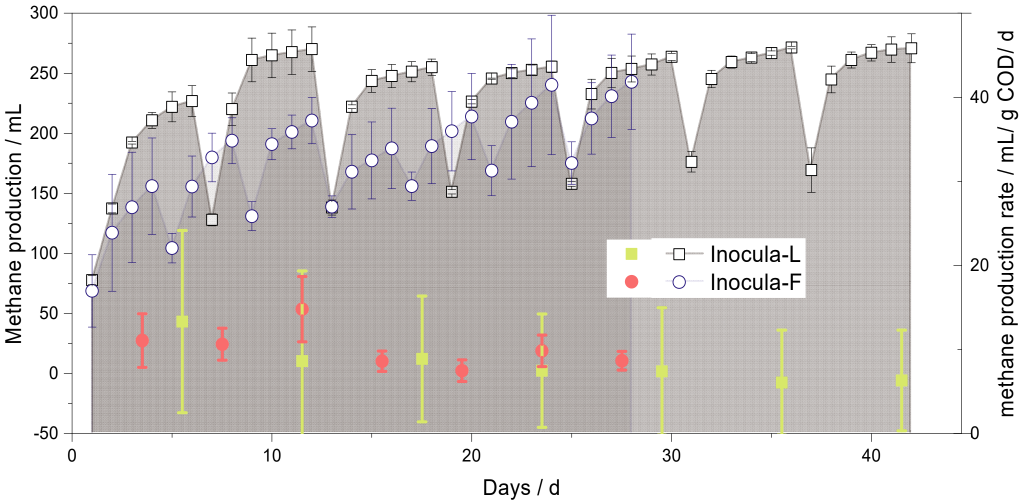

Figure S1 Performance of adaptation stage (Related to Figure 1)

Methane production performance of inocula-F and inocula-L over the stages of adaptation

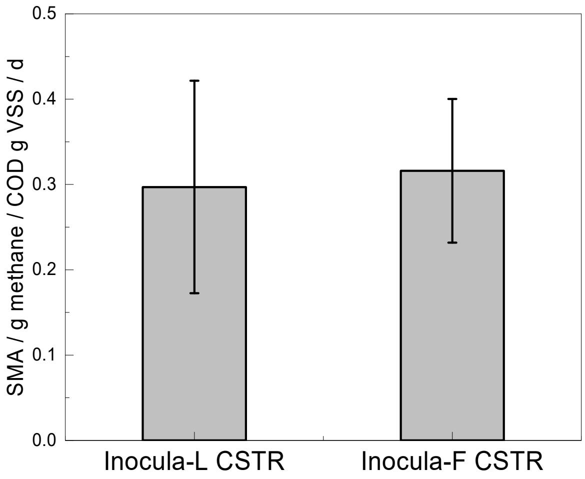

Figure S2 Specific methanogenic activity (SMA) test (Related to Figure 1)
Result of SMA test for inoculum-F and L CSTRs

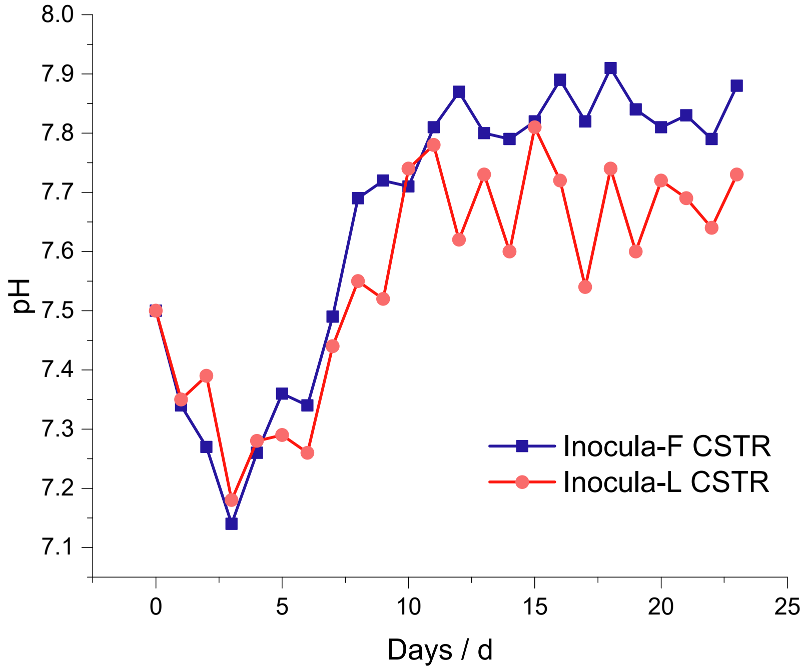

Figure S3 (Related to Figure 1 and Figure 2)

pH variation of CSTRs inoculated with full-scale and laboratory sludge

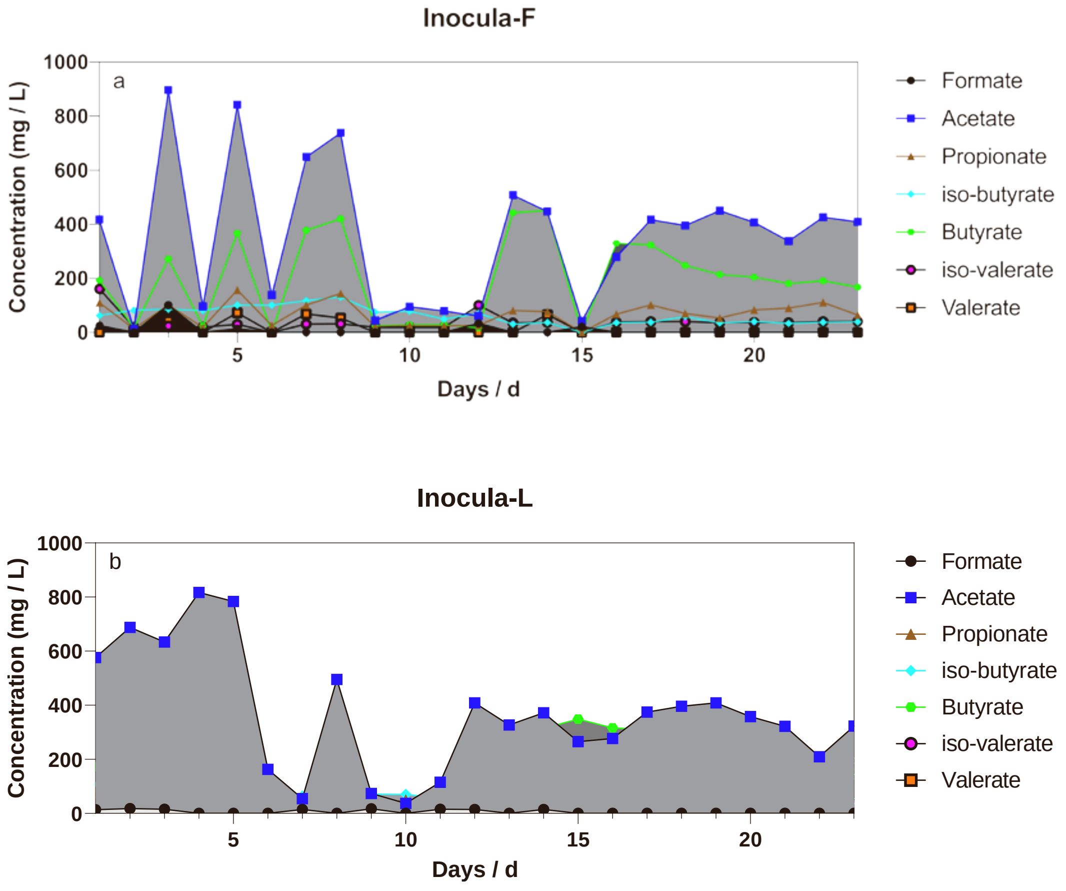

Figure S4 Volatile fatty acids (VFA)variation (Related to Figure 2)

VFA variation in inocula-F (a) and inocula-L (b) CSTRs

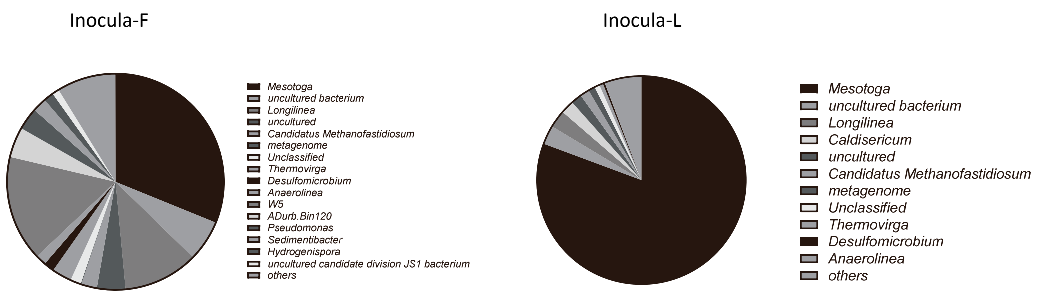

Figure S5 Microbial taxonomy (Related to Figure 3 and Figure 4)

Taxonomic composition of inocula

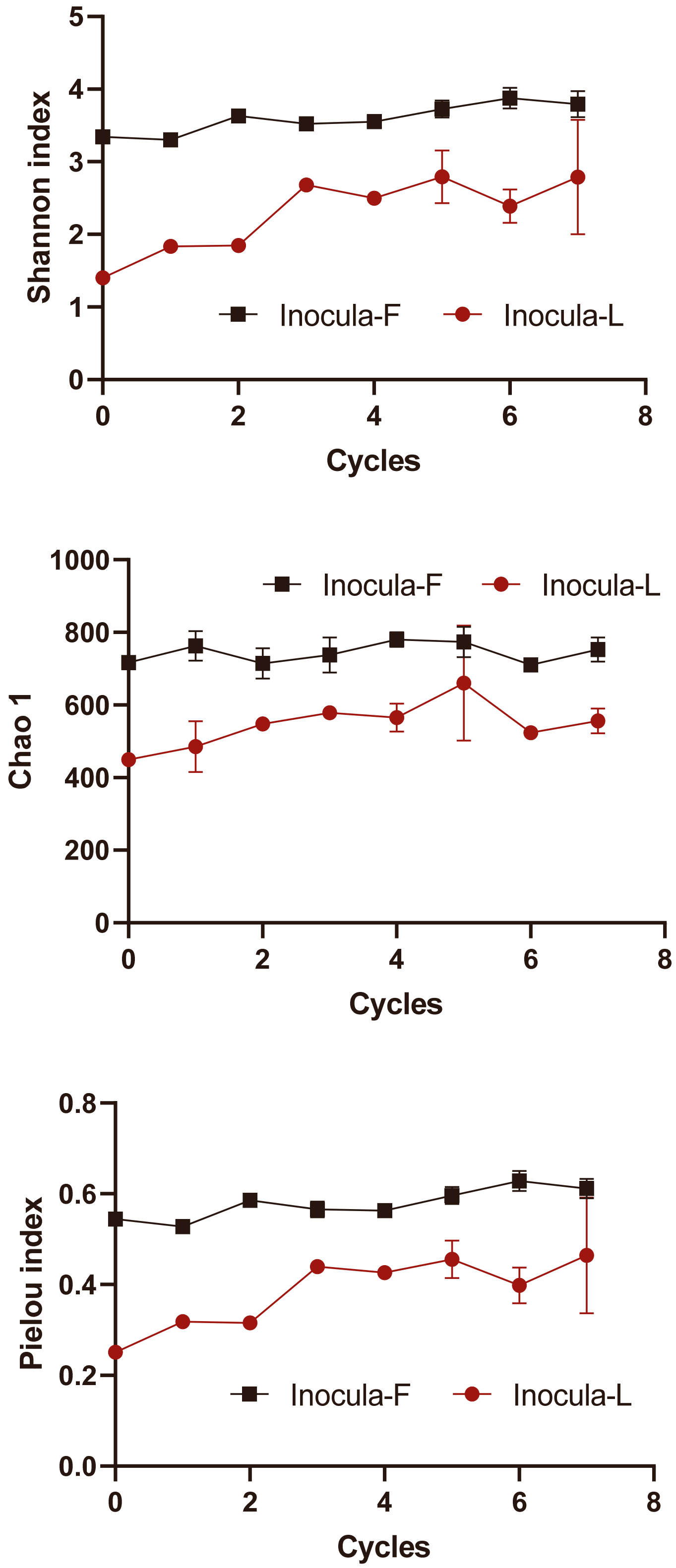

Figure S6 Alpha diversity (Related to Figure 3 and Figure 4)

Diversity index of inocula-F and inocula-L

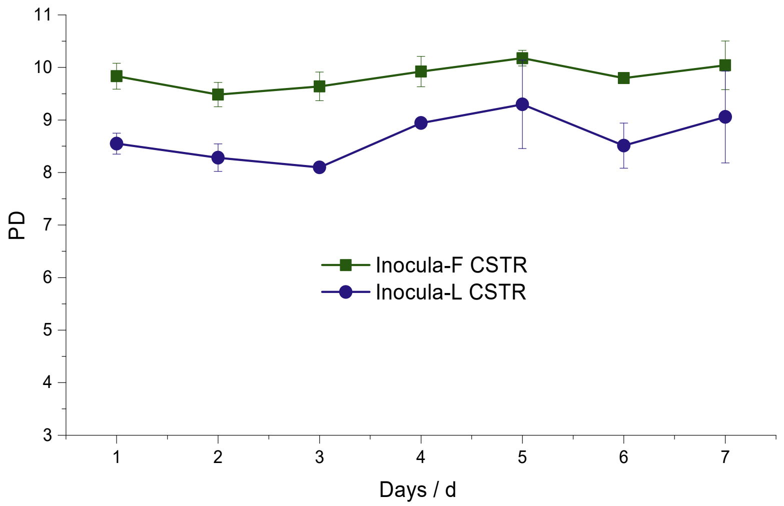

Figure S7 Phylogenetic diversity (PD) of adaptation stage (Related to Figure 3 and Figure 4)
PD variation during the batch adaptation period

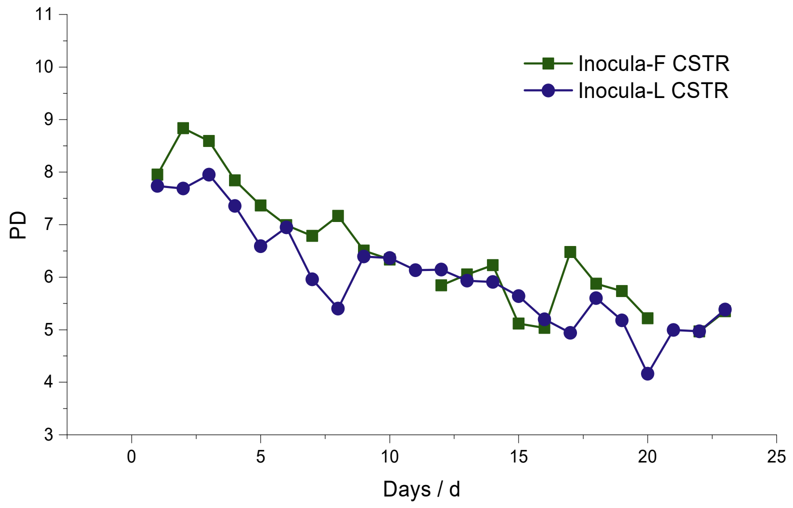

Figure S8 Phylogenetic diversity (PD) of CSTR stage (Related to Figure 3 and Figure 4)
PD variation in CSTR period

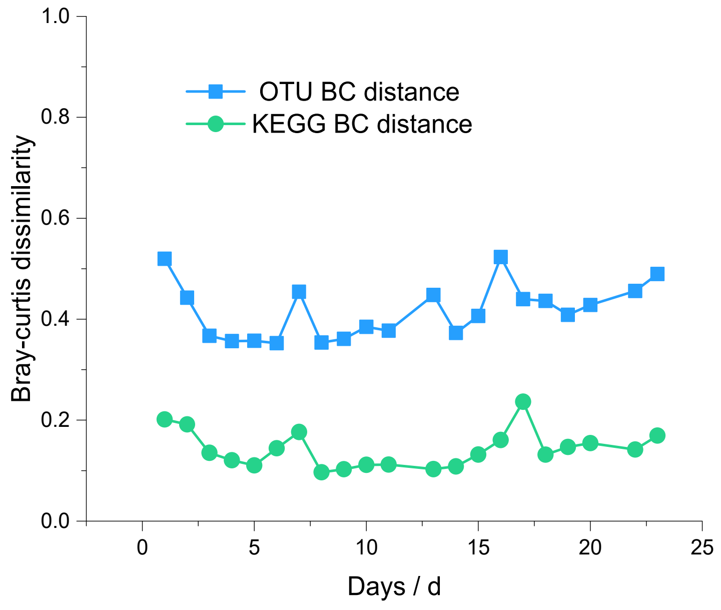

Figure S9 Beta-diversity (Related to Figure 4)

Bray-Curtis dissimilarity based on OTU and KEGG during CSTR operation

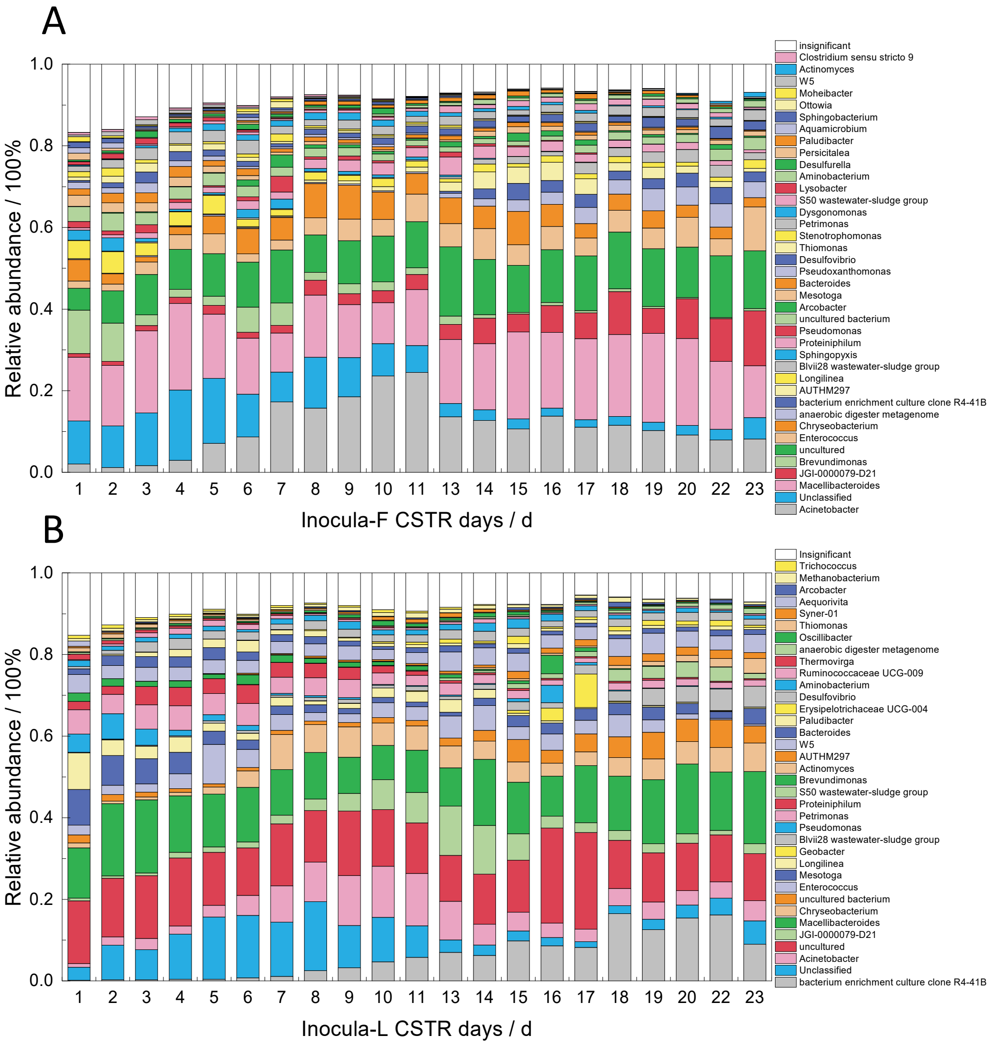

Figure S10 Microbial taxonomy (Related to Figure 5)

Microbial composition at the genus level during CSTR operation (A) inocula-F; (B) inocula-L

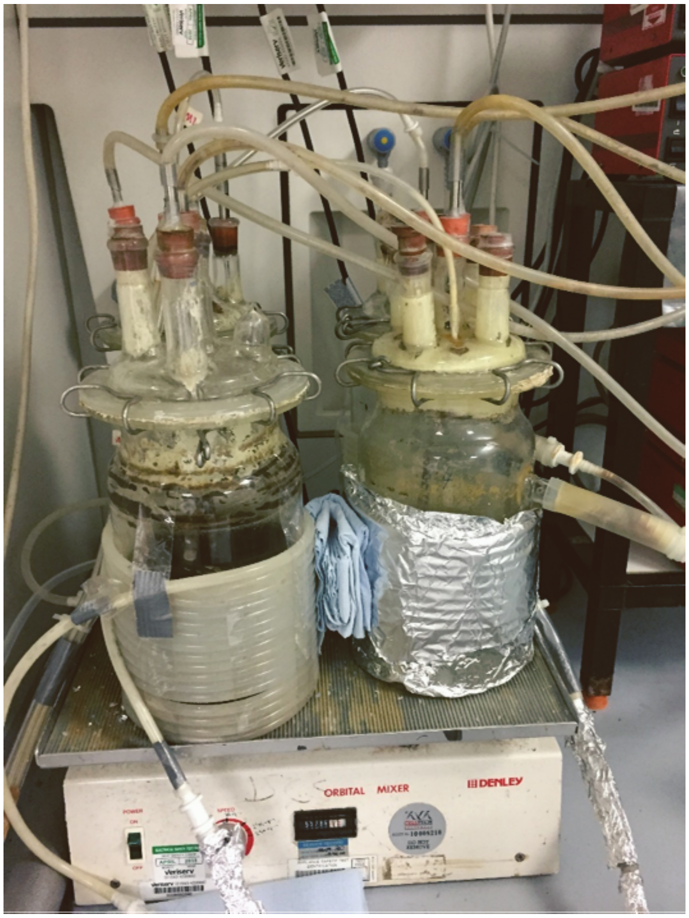

Figure S11 Bioreactors (Related to STAR Method)
The configuration of CSTR

Table S2 Functional prediction (Related to Figure 5)

Functional profiling of specific genus varied significantly over time in Inocula-F and Inocula-L CSTRs

Genus	Inocula-F	Inocula-L	Fermentation	Short-chain fatty acids	Sugars	Proteins/amino acids	reference
Acinetobacter	1	1	-1	1	1	1	Midas
Actinomyces	1	1	1	NA	1	NA	
Aequorivita	NA	1	NA	NA	NA	NA	Aequorivita
Aminobacterium	1	1	1	-1	-1	1	Midas
anaerobic digester metagenome	1	1	NA	NA	NA	NA	
Aquamicrobium	1	NA	NA	1	1	NA	
Arcobacter	1	1	-1	NA	NA	1	
AUTHM297	1	1	NA	NA	NA	NA	
bacterium enrichment culture clone R4-41B	1	1	NA	NA	NA	NA	
Bacteroides	1	1	1	NA	1	NA	
Blvii28 wastewater-sludge group	1	1	1	-1	1	1	
Brevundimonas	1	1	NA	1	1	1	
Chryseobacterium	1	1	-1	NA	1	1	
Clostridium sensu stricto 9	1	NA	NA	NA	NA	NA	
Desulfovibrio	1	1	NA	NA	NA	NA	
Desulfurella	1	NA	NA	NA	NA	NA	
Dysgonomonas	1	NA	1	NA	1	NA	(Lawson et al., 2010)
Enterococcus	1	1	1	1	1	1	Midas
Erysipelotrichaceae UCG-004	NA	1	NA	NA	NA	NA	
Geobacter	NA	1	NA	1	NA	NA	

insignificant	1	1	NA	NA	NA	NA	
JGI-0000079-D21	1	1	NA	NA	NA	NA	
Longilinea	1	1	NA	NA	NA	NA	
Lysobacter	1	NA	NA	NA	NA	NA	
Macellibacteroides	1	1	1	NA	1	NA	(Jabari et al., 2012)
Mesotoga	1	1	1	NA	NA	NA	(Nesbø et al., 2012)
Methanobacterium	NA	1	1	NA	NA	NA	Midas
Moheibacter	1	NA	NA	NA	NA	NA	
Oscillibacter	NA	1	1	NA	NA	NA	FAPROTAX
Ottowia	1	NA	NA	NA	NA	NA	Midas
Paludibacter	1	1	1	NA	1	NA	(Ueki et al., 2006)
Persicitalea	1	NA	NA	NA	NA	NA	
Petrimonas	1	1	1	1	1	1	Midas
Proteiniphilum	1	1	1	1	1	1	
Pseudomonas	1	1	1	NA	NA	NA	(Schreiber et al., 2006)
Pseudoxanthomonas	1	NA	NA	NA	NA	NA	
Ruminococcaceae UCG-009	NA	1	NA	NA	NA	NA	Midas
S50 wastewater-sludge group	1	1	NA	NA	NA	NA	
Sphingobacterium	1	NA	NA	NA	NA	NA	(YABUUCHI et al., n.d.)
Sphingopyxis	1	NA	-1	1	1	1	Midas
Stenotrophomonas	1	NA	NA	NA	NA	NA	(Gautam et al., 2009)
Syner-01	NA	1	NA	NA	NA	NA	Midas
Thermovirga	NA	1	1	-1	-1	1	

Thiomonas	1	1	1	NA	NA	NA	FAPROTAX
Trichococcus	NA	1	1	1	1	Variable	Midas
Unclassified	1	1	NA	NA	NA	NA	
uncultured	1	1	NA	NA	NA	NA	
uncultured bacterium	1	1	NA	NA	NA	NA	
W5	1	1	NA	NA	NA	NA	

1 represents positive; NA represent not available
